# Robustness of the Krebs Cycle under Physiological Conditions and in Cancer: New Clues for Evaluating Metabolism-Modifying Drug Therapies

**DOI:** 10.3390/biomedicines10051199

**Published:** 2022-05-22

**Authors:** Rafael Franco, Joan Serrano-Marín

**Affiliations:** 1 CiberNed, Network Center for Neurodegenerative Diseases, National Spanish Health Institute Carlos III, 28029 Madrid, Spain; 2 Molecular Neurobiology Laboratory, Department de Bioquímica i Biomedicina Molecular, Universitat de Barcelona, 08028 Barcelona, Spain; joan.serrano.marin@gmail.com; 3 School of Chemistry, Universitat de Barcelona, 08028 Barcelona, Spain

**Keywords:** carcinoma, mitophagy, broken Krebs cycle, citric acid cycle, anaplerotic reactions

## Abstract

The Krebs cycle in cells that contain mitochondria is necessary for both energy production and anabolic processes. In given cell/condition, the Krebs cycle is dynamic but remains at a steady state. In this article, we first aimed at comparing the properties of a closed cycle versus the same metabolism in a linear array. The main finding is that, unlike a linear metabolism, the closed cycle can reach a steady state (SS) regardless of the nature and magnitude of the disturbance. When the cycle is modeled with input and output reactions, the “open” cycle is robust and reaches a steady state but with exceptions that lead to sustained accumulation of intermediate metabolites, i.e., conditions at which no SS can be achieved. The modeling of the cycle in cancer, trying to obtain marked reductions in flux, shows that these reductions are limited and therefore the Warburg effect is moderate at most. In general, our results of modeling the cycle in different conditions and looking for the achievement, or not, of SS, suggest that the cycle may have a regulation, not yet discovered, to go from an open cycle to a closed one. Said regulation could allow for reaching the steady state, thus avoiding the unwanted effects derived from the aberrant accumulation of metabolites in the mitochondria. The information in this paper might be useful to evaluate metabolism-modifying medicines.

## 1. Introduction

The discovery of the tricarboxylic acid cycle (TAC) by Krebs was one the main achievements of the 20th century. The discovery of TAC led to (i) the understanding of how eukaryotic cells obtain the energy coming from reduced compounds and (ii) conceptually separating the obtention of reducing power from the use of oxygen and CO_2_ production, both processes being tightly coupled and taking place in the mitochondrion. With few exceptions, e.g., in cells lacking mitochondria, every mammalian cell type has an operative TAC that provides energy in the form of NADH and FADH_2_, which enter into the electron transport chain and the oxidative phosphorylation machinery to produce ATP.

Under physiological conditions, the TAC is not a closed cycle as there are the so-called anaplerotic reactions that provide metabolites of the cycle or, alternatively, that remove them. Anaplerotic reactions can serve to refill the cycle, but also take part in anabolic and catabolic processes. In fact, reactions in the TAC are part of anabolic and catabolic routes. An example is the production of amino acids from TAC intermediates. Accordingly, some of the reactions of the TAC are part of the anabolic routes: from synthesis of proteins to synthesis of amino acid-derived hormones and neurotransmitters.

Whether for good or bad, the TAC plays a role in various pathological conditions. On the one hand, mitochondrial resilience seems to be the basis for the slow evolution of neurodegenerative diseases. On the other hand, altered mitochondria lead to higher risk of suffering from neurodegenerative diseases. The higher risk of Alzheimer’s disease in the progeny of mothers with a family history of the disease seems to be mediated by mitochondria, thus suggesting a mitochondrial inheritance [1,2,3].

The TAC is regaining interest in cancer due to the finding of mutated forms of the enzymes of the TAC that shifts substrate selectivity and leads to the production of oncometabolites [4]. However, reactions in the TAC are key for cancer progression. The fact that cancer cells are generally more dependent on glycolysis than on TAC for energy production is known as the Warburg effect [5]. This view has been recently challenged. It has been reported that lactate may be a fuel used by cells of lung cancer, and that radiolabeled lactate infused to patients results in incorporation of the radioactive label in TAC intermediates [6]. A very recent article shows that the Warburg effect is important in non-dividing intestinal cells, not for energy but to combat oxidative stress [7]. There is convergent evidence pointing to TAC as instrumental in cancer; there is for instance the observation that tumorigenesis is limited when mitochondrial DNA is removed [8]. It is also becoming apparent that tumors are metabolically less homogeneous than previously suspected and that there are cells in the tumor that rely less on glycolysis than on aerobic TAC-mediated energy production [9]. The need for building blocks for protein and nucleic acid synthesis in proliferating cancer cells depends to a greater or lesser extent on the availability of TAC intermediates and the TAC operation to produce sufficient ATP.

A recent paper has used the queuing theory to model the TAC in such a way that the dynamics of changes in the concentration of TAC intermediates in response to any alteration in enzyme activities is characterized in silico; for instance, by modeling enzyme inhibition or how fast the cycle can reach the stability [10]. A question that has not been fully answered is the performance of the cycle under homeostatic conditions, in which energy production and anabolic and catabolic processes are in an SS.

The aim of this work was to better understand the operation of TAC in physiological conditions and in cancer, always assuming a dynamic SS or asking whether the SS was possible upon varying parameter values, individually or in combination. Parameters describing an SS will vary from cell type to cell type and also from a given condition to another condition, physiological or pathological. SS is defined in this case as having a constant flux through the reactions in the cycle and constant intermediate concentrations. The results show that the TAC is very robust except in conditions that do not allow for attainment of an SS. We also show that the major changes in energy production by the TAC in cancer are consistent with a limited Warburg effect.

## 2. Materials and Methods

The in silico Krebs cycle model, developed in the late 1980s using FORTRAN programming language, is used as reference [11]. After obtaining the general design of the aforementioned publication, we proceeded to obtaining simpler equations and collecting more faithful variables by exchanging Kms obtained from non-human enzymes for Kms obtained from human samples or updating some values measured by more modern methods.

The equations were similar to those in Canela et al. (1987) [11], adapted to take advantage of equations provided by COPASI, a free software application for simulation and analysis of biochemical networks and their dynamics. See Equations in the Supplementary Information for details. The values of the parameters were mainly taken from data available in BRENDA database. The Human Metabolome Database was used for obtaining the metabolite concentrations. Although COPASI allows for creating models involving different compartments (cytoplasm, periplasmic space, mitochondria, nucleus), a single compartment was used in the model here addressed.

**Figure 1:**
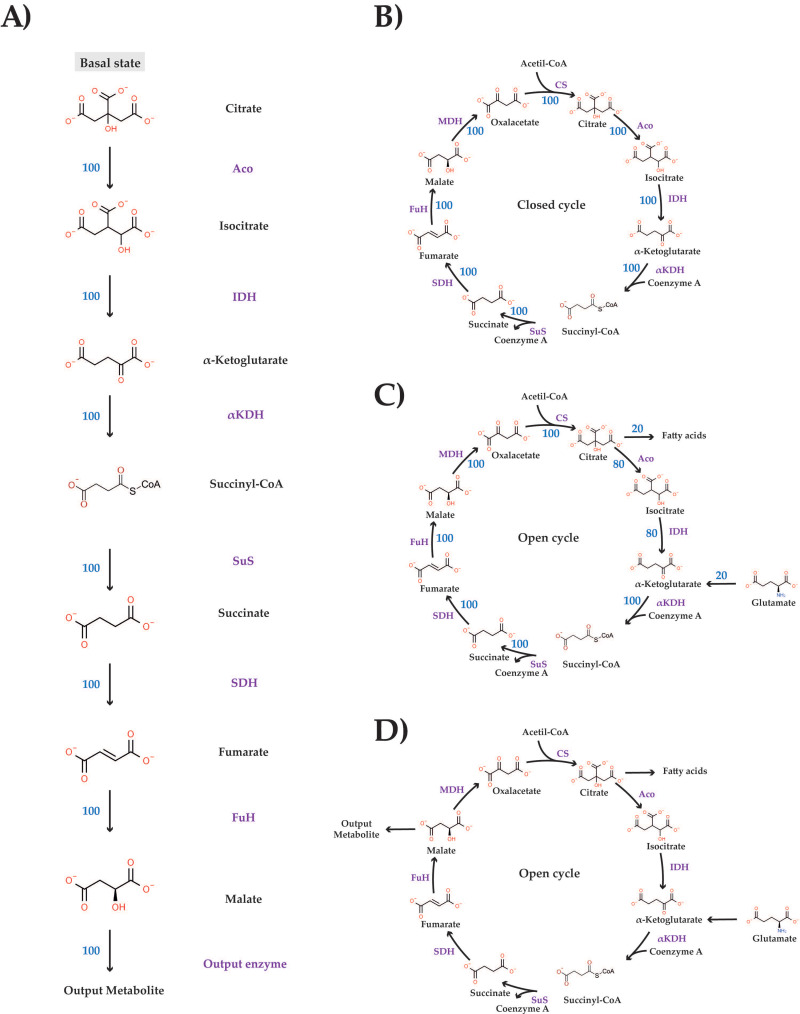
**Models.** (**A**): **Linear-like Krebs’ cycle.** The numbers in each arrow indicate the flux through every reaction (relative units but simulated as if they were in mmol/s). (**B**): **Closed Krebs’ cycle.** (**C**): **Open Krebs’ cycle.** One input (IF) and one output (OF) fluxes. (**D**): **Open Krebs’ cycle.** One input and two output fluxes. Abbreviations are: CS: citrate synthase (v1); Aco: aconitase (v2); IDH: isocitrate dehydrogenase (v3); αKDH: α-ketoglutarate dehydrogenase (v4); SuS: succinyl-CoA synthetase (v5); SDH: succinate dehydrogenase (v6); FuH: fumarate hydratase (v7); MDH: malate dehydrogenase (v8). The number near every arrow corresponds to the flux in initial (control) conditions.

Unless otherwise stated, the metabolism is studied at a steady state (SS), which requires metabolite concentrations and fluxes to be constant. In the initial SS, the flux (in relative units) was fixed to 100 both when modeling a TAC-like linear metabolism (Figure 1A) and when modeling a closed TAC (Figure 1B). In the open cycle, the input flux (IF) is 20 and the output flux (OF) is 20 (Figure 1C); hence, fluxes trough the catalytic steps of the cycle were 100 and 80; 80 for aconitase and isocitrate dehydrogenase steps and 100 for the remaining ones.

For the time-course simulation, a time-course assay has been made by the deterministic (LSODA) simulation method, which is a differential equations solver. This solver is able to find the solution of differential equations systems with a dense or banded Jacobian method when the conditions are stiff, but it automatically selects between non-stiff, Adams, and stiff, backward differentiation methods. The program uses the Adam method initially, but there a dynamical check of data at every integration step to decide which method to use in the next step [12]. The relative tolerance, which was used as an estimator of the simulation accuracy, was set to 1.0 × 10^−6^ (note that the minimum relative tolerance is 2.2 × 10^−16^). The absolute tolerance was 1.0 × 10^−12^.

## 3. Results

### 3.1. Steady-State Conditions

The TAC is a dynamic process that in a given condition, a cardiomyocyte in a heart with sinus rhythm and a given number of beats/min, a hepatocyte in fasting conditions, a resting microglial cell, a proliferating cancer cell, etc., is in steady state, i.e., both the flux through the cycle and the concentration of TAC metabolites remain constant. Accordingly, and unless otherwise stated, the TAC will be here considered as in a steady state (SS) or dynamically changing to reach a new SS.

### 3.2. Effect of Enzyme/Metabolite Level Variation in a Linear Version of the TAC

Although the TAC is indeed a cycle, it is of interest to know what its operation would be if it were a linear metabolic pathway. The linear version of the TAC here tested is shown in Figure 1. It was handled as a linear metabolism as considered by the Metabolic Control Theory (MTC) [13], and in SS. The intracycle (IC) flux is arbitrarily set to 100, but the numerical value of the IC flux does not affect the results, that is, for any value of the IC flux in SS conditions, the results would be qualitatively the same. In other words, flux variations will not affect the qualitative results nor the intermediate concentrations in SS. Accordingly, we have taken metabolite concentrations from in the literature (Table 1) and the equations for each enzymatic step (see Equations in Supplementary Material) to calculate the V_max_ values, providing a flux (enzymatic rate/activity) of 100 in each step. First, we have perturbed this SS (flux = 100 and metabolite concentrations provided in Table 1) varying either the level/catalytic activity of enzymes or the concentration of intermediates. After every perturbation, simulation is performed until an SS is achieved or when it is evident that a new SS cannot be achieved. As described later, there are conditions that impede obtaining an SS.

Upon a two-fold increase or decrease in the α-ketoglutarate concentration, the IC flux is the same as the initial one (100), and the metabolite levels are the same as at the beginning of the simulation (Table 1), that is, the system returns to the same SS. We repeated the simulation but increasing or decreasing the α-ketoglutaric acid concentration by two orders of magnitude and the results were the same. Variations of any other metabolite led to fairly identical results. It should be noted that citrate is not considered a variable because its concentrations is fixed, i.e., keeping the IC flux constant requires to keep the concentration of citrate constant. As indicated of Figure 1D, the production of the “output metabolite” by the “output enzyme” would be a measure of the flux that is exiting in this linear metabolism.

**Figure 2:**
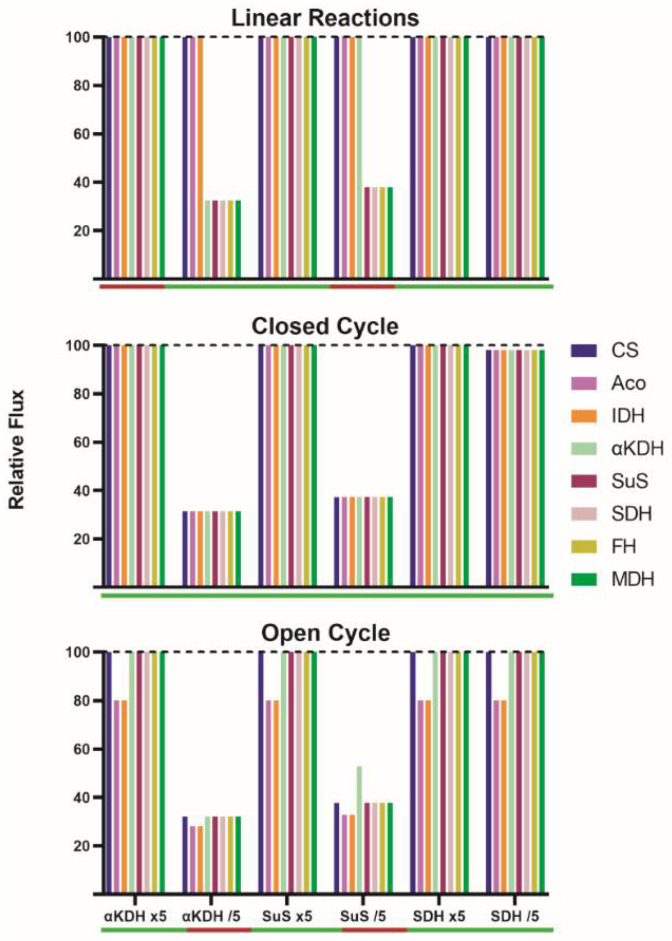
Histograms showing the variation in the relative flux through each TAC step in different conditions. The dotted line indicates the IF of 100 in the initial SS. The red/green horizontal bar under each condition indicates if an SS is reached (green) or not (red). When SS is not reached there are intermediates whose concentrations change with time (See Figure 3 for data derived from considering the closed and open TAC).

A 5-fold increase in the nominal catalytic activity of α-ketoglutarate dehydrogenase (v4), succinyl-CoA synthetase (v5) or succinate dehydrogenase (v6), did not change IC flux (Figure 2) but metabolite concentrations (Supplementary Table S1). Even simultaneously increasing the activity of enzymes in v4 and v6 (or in v4 and v5), the flux after stabilization was the same as in the initial SS. Changes in steps 4, 5, or 6 led, in the new SS, to significant variations in the concentration of α-ketoglutarate, succinyl-CoA, and succinate. As expected, those concentrations also varied when the activity of two enzymes increased simultaneously (Figure 2, Supplementary Table S1). For each condition, the flux was the same as the initial one (100) because the concentration of each metabolite was adjusted to a level that provides a catalytic activity of 100; depending on the condition, the concentration of each metabolite in the new SS may increase or decrease (Supplementary Table S1). When stabilization was reached, the flux through each metabolic step in each of the conditions was as shown in Figure 2.

The decrease in the activity of some of the enzymes caused drastic changes. First, a control was performed assuming a reduction in the activity of succinate dehydrogenase (v6); such a change should not affect the IC flux because it is in the last part of the pathway and the enzyme does not have a significant flux control coefficient. In fact, the flux in the new SS was the same as in the initial one. However, a 5-fold reduction in α-ketoglutarate dehydrogenase activity (v4) led to a reduction in the flux though steps catalyzed by α-ketoglutarate dehydrogenase, succinyl-CoA synthetase, succinate dehydrogenase, fumarase and malate dehydrogenase; in all these steps the flux went from 100 to 32.5. Consequently, if some reactions proceed at a rate of 32.5 and the rest at a rate of 100, no new SS is possible.

A 5-fold reduction in succinyl-CoA synthetase activity (v5) led to a flux of 37.8 in steps catalyzed by succinyl-CoA synthetase, succinate dehydrogenase, fumarase and malate dehydrogenase. When the activity of α-ketoglutarate dehydrogenase and succinate dehydrogenase were simultaneously decreased (by five-fold), the enzymatic activity in every step was the same as that found when a reduction in α-ketoglutarate dehydrogenase activity was simulated (Supplementary Table S1).

In several conditions by which enzyme activities are modified, the concentration of some metabolites changed irrespective of whether the output flux in the new SS was as in the initial SS (Supplementary Table S1). The most striking variations in concentration were observed when activities were reduced. A 5-fold reduction in the activity of some of the enzymes caused variations of two orders of magnitude in some metabolite concentrations. In addition, there were cases that would unlikely be found in a physiological situation, such as the 5-fold decrease in α-ketoglutarate dehydrogenase that leads α-ketoglutarate going from 0.32 to 67501, i.e., >5 orders of magnitude higher (Supplementary Table S1).

Taken together, these results confirm that in a linear metabolism, any variation in the concentration of metabolic intermediates would not lead to any change in SS, that is, over time, the new SS would be indistinguishable from the initial one. In contrast, variation in enzyme catalytic activities can lead to substantial changes. From a given SS, increases in enzyme activities do not lead to flux variations, whereas the decrease in the activities of key enzymes, those with higher flux control coefficient, would lead to a decrease in the production of the “output metabolite”, but more importantly, to the accumulation of some intermediates, meaning that no SS could be attained.

### 3.3. Effect of Enzyme/Metabolite Level Variation in a Closed TAC

The closed version of the TAC is shown in Figure 1B. The flux is arbitrarily set to 100, but the numerical value of the flux does not affect the qualitative results nor the intermediate concentrations in SS or through dynamic processes. Accordingly, we have proceeded to calculate the V_max_ leading to an IC flux of 100 using the metabolite concentrations displayed in the literature (Table 1) and the equations for each enzymatic step indicated in Supplementary Material. This will be considered the reference condition or “control” TAC. Subsequently, we perturbed this SS (enzyme rate/activity for each TAC step set to 100) and metabolite concentrations given in Table 1 by varying the catalytic level/activity of the enzymes or the concentration of metabolic intermediates. After every perturbation, the simulation was performed until an SS was achieved either “returning” to the initial one or “going” to a new SS. It is anticipated that in a closed TAC, the SS can always be achieved.

**Figure 3:**
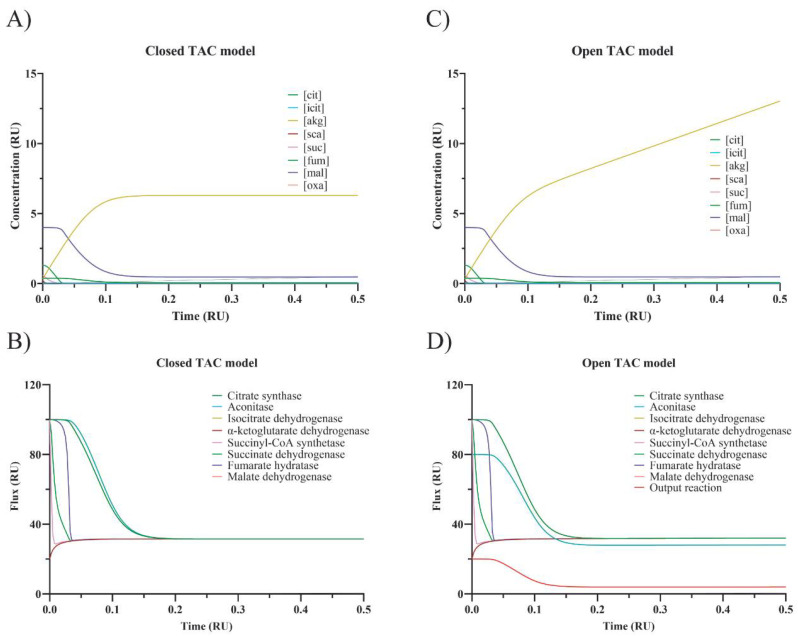
Dynamic variation of flux trough every step and of intermediate concentrations in the closed and open TAC when V4 (α-ketoglutarate DH) is reduced by 5-fold. The closed cycle (panels (**A**,**B**)) refers to that depicted in Figure 1B and the open cycle (panels (**C**,**D**)) to that depicted in Figure 1C. RU: relative Units.

First, a 5-fold increase in the nominal activity of any enzyme in the closed TAC leads to minimal changes in the IC flux in the new SS (less than 2% variation) (Supplementary Table S2). A much lower flux, but still constant throughout the closed cycle, is obtained when decreasing by 5-fold the activity of α-ketoglutarate dehydrogenase (from 100 to 31.5) or of succinyl-CoA synthetase (from 100 to 37.3) (Supplementary Table S2). Interestingly, a reduction in succinate dehydrogenase activity provokes a minimal IC flux decrease (from 100 to 98.1). When the activity of two enzymes is simultaneously decreased by 5-fold, the IC flux is not decreased in an additive manner. Regardless of the perturbation, an SS is achieved, implying equal enzyme rates at each step of the closed TAC. What changes for each new SS are the concentrations of metabolites to fit the new condition. A change in enzyme level/activity leading to small changes in the flux in the new SS leads to small variations in metabolite concentrations in the new SS (Supplementary Table S2). This finding occurs irrespective of the enzyme whose activity is modified. Surely, any change in enzyme activity led to local variations of metabolite concentrations. For instance, an increase (×5) in succinate dehydrogenase leads to small variations, except for in succinyl-CoA (from 0.23 to 0.15), succinate (from 0.57 to 0.22) and fumarate (from 1.3 to 1.6). When the activity of α-ketoglutarate dehydrogenase is increased (×5), significant variations occur in the concentration of α-ketoglutarate (from 0.32 to 0.028) but not in the other compounds (range of variation: 0–20%). In contrast, metabolite concentrations may markedly change in conditions leading to SS fluxes between 30 and 40 (See Supplementary Table S2). A decrease (/5) in α-ketoglutarate dehydrogenase causes the citrate, isocitrate, α-ketoglutarate, succinyl-CoA, succinate, fumarate, malate and oxaloacetate concentrations in the final SS be, respectively, 14, 31, 1965, 7, 0.2, 0.6, 12 and 2%, (percentage respect to values in the control TAC), i.e., the level of all metabolites, but that of α-ketoglutarate decreases. The SS achieved when α-ketoglutarate dehydrogenase activity is reduced 5-fold is shown in Figure 3 (panel A for fluxes through every step and panel B for intermediate concentrations).

Let us now consider how a variation in metabolite concentrations would affect the flux and/or the concentration of other metabolites. Varying the concentration of α-ketoglutarate (×2 or /2) there were no major variations in the concentrations of metabolites when the system stabilized and the flux in the new SS was modified by a modest 0.2%. We forced the system by increasing or decreasing the level of the compound by two orders of magnitude (×100 and /100). Even so, the flux varied by less than 3%, although there were significant changes in the level of some metabolites, mainly when compound level was increased. Increasing the concentration of α-ketoglutarate from 0.32 to 32 led to changes in succinyl-CoA (2.3-fold increase), succinate (37-fold increase), and fumarate (5.2-fold increase), while malate and oxaloacetate were mildly increased. It should be noted that α-ketoglutarate itself went from 32 at the beginning of the simulation to 0.34, which is similar to the value in the initial SS (0.32). Qualitatively similar results were obtained by increasing or decreasing the concentration of succinate by two orders of magnitude. These results indicate that the TAC is robust and major perturbations in metabolite concentrations leads to rebalancing the whole system in such a way that the maximal variation of the flux in SS was less than 3%.

Remarkably, one of the conclusions derived from these results is that the closed cycle is always able to reach a “new” SS, either the same as the original one or another; none of the modifications lead to gross alterations in the flux through TAC steps.

### 3.4. Effect of Enzyme/Metabolite Level Variation in a TAC with Anaplerotic Steps (“Open” TAC)

The occurrence of anaplerotic routes that refill the TAC and the use of some metabolites of the cycle for biosynthetic purposes are well-known. Accordingly, we modelled the cycle depicted in Figure 1C, in which the cycle has an input flux (IF) at the level of α-ketoglutarate and an output flux (OF) at the level of citrate. With only one input reaction and one output reaction, any SS requires that the input reaction flux (i.e., IF) be the same as the output reaction flux (i.e., OF). Hence, we assumed that the IF and OF were equal to 20 (in arbitrary units). Hence, the enzyme activity for the reactions between α-ketoglutarate and citrate was set at 100 and the flux through the remaining reactions was set at 80 (see Figure 1C).

It was first noticed that a 5-fold increase in the nominal activity of any step, or even of two steps altogether, leads to unmodified fluxes in the “new” steady state (less than 2% flux variation, Supplementary Table S3). A 5-fold decrease in the activity of succinate dehydrogenase does not significantly modify the fluxes (100 and 80). Those conditions that lead to unchanged 100/80 fluxes or to slightly modified fluxes may lead to significant changes in concentrations, but limited to 1-2 metabolites. When catalytic activities were increased (5 times), the main change is in the concentration of the substrate or in the concentrations of substrate and product of the enzyme whose activity was modified. The decrease in succinate dehydrogenase, which does not lead to any variation in fluxes, provoked an increase in both in succinyl-CoA (from 0.23 to 1.02 mM) and succinate (from 0.57 to 3.96 mM) concentrations (Supplementary Table S3).

Interestingly, it was discovered that some conditions did not allow for obtaining a new SS. A 5-fold decrease in α-ketoglutarate dehydrogenase led to a significant reduction in both the flux between α-ketoglutarate and citrate (from 100 to 32.2) and the flux through the remaining steps (from 80 to 28.2). The decrease was also marked in the case of decreasing succinyl-CoA synthetase activity (from 100 to 37.8 and from 80 to 32.8). The simultaneous decrease in the activity in these two steps led to similar reductions (from 100 to 32.5 and from 80 to 28.5). These results suggest that the cycle may be self-regulated. However, self-regulation does not lead to any SS in some of the conditions that were simulated. For instance, the continuous entrance of carbons via α-ketoglutarate combined with a reduction in the flux in some of the steps leads to a continuous increase in the level of some intermediates. A 5-fold decrease in v4 impedes SS with a sustained accumulation of α-ketoglutarate. A 5-fold decrease in v5 impedes SS with a sustained accumulation of succinyl-CoA. If the simulation continues, the increase in concentration may be of several orders of magnitude, that is, these conditions cannot be maintained for long and/or are not physiological (see Supplementary Table S3). The lack of new SS when α-ketoglutarate dehydrogenase activity is reduced 5-fold is shown in Figure 3 (notice the sustained increase in α-ketoglutarate dehydrogenase in panel D).

In this “open” model of TAC, which is more physiological than a closed TAC, the change in α-ketoglutarate concentration does not lead to any change in fluxes, which remain 100 and 80. Even when changing an intermediate concentration by two orders of magnitude, stabilization occurs when the original flux values (100 and 80) are reached. Furthermore, concentration variations were completely buffered by the system that, upon stabilization and SS attainment, displayed the same values in fluxes and concentrations as in the initial SS (Supplementary Table S3).

### 3.5. The TAC in Proliferating Cancerous Cells

The Warburg effect is mainly characterized by a decrease in energy production by the TAC of the proliferating cancerous cell. Considering the robustness of the cycle and the above-described results, there are different options for reducing the flux of the TCA (the open cycle was selected). A major disequilibrium in the ratio of fluxes may occur, for instance, if the activity of MDH_m_ is reduced by one order of magnitude (90% reduction in activity in the original SS), the OF goes from 20 to 1.7. This cannot be physiological, as the OF, which produces the compound needed to fatty acid synthesis (citrate), should not change much in cancer cells. In fact, together with the marked reduction in the OF, no SS can be reached because malate accumulates; therefore, to maintain stability in the system, a withdrawal reaction to take out malate from the cycle would be needed. A similar reduction in citrate synthase activity (90% reduction in activity in the original SS), which mimics a shortage of acetyl-CoA, also reduces OF needed for fatty acid synthesis (from 20 to 1.7, i.e., similar to that found after a one-order of magnitude reduction in MDH_m_ activity). Again, a low OF is accompanied by malate accumulation and no SS can be achieved unless a withdrawal reaction is built into the model to remove malate from the cycle. When both MDH_m_ and citrate synthase activities are simultaneously reduced to a 20%, the flux is not reduced as much as when reducing MDH_m_ activity to 10%; however, the OF is still very low (3.7) and no SS is achieved. Adding a malate withdrawal reaction could be enough to obtain the SS, although a condition must be fulfilled, namely that the sum of the two OF is equal to the IF (20). This condition must be fulfilled regardless of whether the activity of one, MDH_m_, or of the two enzymes, MDH_m_ and citrate synthase, are modified. Then, we have modified the cycle to consider an extra withdrawal at the level of malate (Figure 1D). Using the TAC modeled as in Figure 1D, more conditions were simulated while trying to keep the OFs as high as possible (OF from malate and OF from citrate). There are conditions that make a new SS possible, but in these cases, for example, by setting the OFs to 80 (instead of 20), the final steady state is reached when the two OFs are equal to the IF (20). The highest reduction in the flux was obtained by a 2-fold reduction in citrate synthase activity, which is equivalent to reducing acetyl-CoA entrance by 2. The reduction in flux is moderate: to 53.2 in those steps whose enzymatic activity was 100 and to 33.2 in those steps whose enzymatic activity was 80; the absolute magnitude of the reduction is the same: 46.8 (100 − 53.2 = 46.8 and 80 − 33.2 = 46.8), thus leading to approximately one-half the production of NADH/FADH_2_ in the new SS. In conclusion, the inertia of the TAC impedes marked reductions in the flux through the cycle.

## 4. Discussion

Mitochondria are at center stage in a variety of diseases. In the late 1980s and the 1990s, alterations in mitochondrial function and in the level of enzymes of the Krebs cycle were associated with neurodegenerative diseases, Parkinson’s and Alzheimer’s included [18,19,20,21,22,23,24,25]. More recently, epidemiological, pharmacological and genetic studies have confirmed that mitochondrial abnormalities are a probable cause in some cases of Alzheimer’s disease and in familial cases of Parkinson’s disease [1,3,26,27,28,29,30,31,32,33,34,35,36,37,38,39,40,41,42,43,44]. When mitochondrial resilience begins to fail due to aging or hereditary factors, sensitive cells, such as neurons, initiate progressive neurodegeneration. In our opinion, the design of biomedicines to combat certain diseases must take into account the mitochondria and their main biochemical process, the Krebs cycle.

Biochemical reactions taking place in living organisms operate far from the equilibrium but under homeostatic conditions. This is reflected by dynamic systems acting in SS in such a way that changes from one circumstance to another, for instance a resting muscle versus the same muscle in exercise, are characterized by parameters defining their respective SS. In this scenario, the TAC is better studied in SS conditions. We already took this approach to show, in 1985, that the purine nucleotides cycle, in muscle and via fumarate, refills TCA and significantly increases the flux during exercise [11]. At that time, the metabolic control theory [13,17] was a valuable tool to study metabolism. Nowadays, the study of metabolism relies on system biology approaches and potent methodologies that allow for quantitation of fluxes and metabolite concentrations [45,46]. We postulate that taking an SS as a reference is mandatory to analyze the large amount of data that can now be generated.

Despite the enzyme concentrations being the independent variables of a given metabolic system, the metabolite concentrations, i.e., the dependent variables, allow us to precisely define the metabolism. In terms of handling the TCA, we have opted for comparing it with a system with the same reactions but in a linear arrangement. The results are similar in that any perturbation in metabolite concentration is buffered by the system. This verification is a further confirmation that the independent variables (enzyme activities) determine the system. In fact, when the system is perturbed by modifying the concentration of any metabolite, even increasing its levels by two orders of magnitude, the system buffers the alteration, and fairly soon, returns to an SS that is equal to the original one. Surprisingly, here we report the conditions by which if we modify some of the independent variables (enzyme activities) we observe that a linear system does not reach the steady state while a closed TAC does; the steady state in the closed TAC is equal to or similar to the basal (control) one. The open TAC operates like the closed TAC but only if the IF is similar (or equal) to the OF. The open TAC can absorb transient disturbances, but cannot reach any SS if differences between nominal IF and OF values are sustained for a long time. Table 2 shows which conditions, shown as those summarized in Supplementary Tables S1–S3, lead to SS or not; only the closed cycle leads, regardless of the disturbance, to a steady state, the same as the original or a “new” one (Table 2).

The robustness of the cycle observed in a theoretical physiological setup is also evident in conditions that are likely occurring in cancer. However, this robustness questions the extent of the Warburg effect, which predicts a reduction in the production of ATP due to a reduction in the production of NADH and FADH_2_ by TAC. We show that it is impossible to markedly reduce the flux unless the availability of acetyl-CoA is markedly reduced or the activity of citrate synthase is downregulated (these two conditions are equivalent because the reaction rate of citrate synthase is proportional to [Acetyl-CoA]). It is not expected that cancer cells suffer from a shortage of the main regulator of TAC activity, ADP, because cancer cells are very active in producing ADP from ATP consumption. A decrease in MDH_m_ leads to slowing down of the cycle, but it is not conceivable that MDH activity is reduced, as citrate, an inhibitor of the enzyme, is used by cancer cells to generate fatty acids. In addition, such a reduction in MDH_m_ activity does not lead to an SS as the concentration of malate steadily increases. A new SS would require withdrawal of malate, but the cycle would be operating without significant changes in flux between citrate and malate. Accordingly, the first step, conversion of oxalacetate to citrate, is likely determining the SS flux of the TAC in the cancer cell. Of interest is the possibility that variations in TAC enzyme activity in cancer cells may lead to mild decreases in flux. This would fit with a scenario in which intermediates of the cycle are used for biosynthetic purposes without drastic changes in the amount of NADH/FADH_2_ generated by the cycle. In summary, what the results tell us is that the reduction in flux and subsequent NADH/FADH_2_ formation and ATP synthesis would not be as high as Warburg predicted. The robustness of the cycle limits the Warburg effect [5], our conclusions, therefore, are in agreement with the views that cancer cells require ATP produced by TCA and oxidative phosphorylation to maintain energy demands for anabolic purposes [8,9,47,48].

Our results may provide a rationale for the results obtained using a label-free optical approach to investigate the redox state of mitochondria and cytosol. The method, which is based on the differential fluorescence of oxidized versus reduced nicotinamide nucleotides, has been used to identify discrete cytosolic and mitochondrial regions in a human breast cancer cell line. The so-called “metabolic clusters” in the mitochondria are areas with similar metabolic activities whose change may be measured. Authors report that deregulation of a Krebs cycle enzyme, ACO2 aconitase, which would theoretically lead to a decrease in the mitochondrial activity of cancer cells, is associated with the activation of mitochondrial metabolism, also involving an increase in electron transport chain operation [49].

The classical view of a substantial Warburg effect in cancer is now reshaped by considering the Krebs to be “broken”, that is, there is a kind of uncoupling of anabolic activity using the TAC intermediates and NADH/FADH_2_/energy production [50,51]. However, how would be an SS achieved in such a broken cycle? We show that the only way is by retrieving intermediates, a possibility that is accompanied by a drastic reduction in the TAC flux. This scenario of removal of metabolites for anabolic reactions but reduction in energy production by the TAC is difficult to understand because glycolysis probably does not produce sufficient amounts of ATP for the anabolic needs of cancer cells. May the TAC be regulated by unknown mechanisms? It is considered that TAC regulation is exerted by allosteric interactions and the metabolic status in terms of NAD/NADH ratios and ADP availability. In other words, it is considered that the hormonal regulation that controls glycolysis/gluconeogenesis (in the liver) [52] has no equivalence in TAC regulation [53]. Marked changes in enzyme activity are not possible in the absence of hormonal (or hormonal-like) regulation. Therefore, the use of TAC intermediates for anabolic purposes, as assumed in a broken Krebs cycle, would deplete the cycle very soon, thus stopping anabolism but also making substantial energy production impossible. Anabolism is possible if the cycle is fully operational (open but operational) and if IF and OF are similar (or equal). The exhaustion of the intermediates or their replenishing more than necessary, that is, unbalancing the system, would prevent the acquisition of an SS. What our model predicts is that, in cases of imbalance, the best option to return to stability is to close the cycle, that is, to halt anaplerotic reactions; a closed cycle would very soon acquire an SS. In short, the broken cycle could not persist if the cycle is not closed: firstly, because it is not possible to obtain a TAC flux close to zero, and secondly, because obtaining a flux close to zero would lead to an energetic failure of the whole system. Are we missing a hormonal-like regulation? Interestingly, this possibility was suggested several decades ago [54], only to be latter forgotten (apparently). The regulation, which was suggested in 1987, was based on calcium as a second messenger [54], an ion that inside the mitochondria acts as an allosteric modulator of some of the enzymes of the cycle [55,56]. It should be also noted that the precursors of mitochondria, bacteria, are regulated by external stimuli/nutrients [57]. In our opinion, the question of whether the cycle can quickly go from open to closed and vice versa needs to be approached carefully to better understand a cycle that everyone knows but that still hides secrets.

## 5. Conclusions

Many mammalian cells depend on the Krebs cycle for key metabolic purposes. Furthermore, mitochondria are attracting interest in a variety of diseases. Consequently, to better design biomedicines that engage mitochondria, a better understanding of the Krebs cycle is needed. First of all, the TAC should be better studied under SS conditions. The dynamics can be complementary but only if the initial and final SS are known. Our results show that the TAC is robust and that the Warburg effect is less marked than initially suggested, since proliferation in cancer cannot lead to marked intracycle flux reductions. We also show that only the closed cycle is stable, and under disturbances, always reaches the steady state (SS). It is tempting to speculate whether, in addition to known Krebs cycle regulation, hormone-like mechanisms could convert an unstable open cycle (unable to achieve any SS) to a stable closed cycle. In other words, an “open” cycle must be tightly regulated in order to reach a SS.

## Figures and Tables

**Table 1:** Metabolite concentrations (in mM) used to model TAC.

Metabolite	Concentration (mM)	Reference
Acetyl-CoA	0.61	[14]
Citrate	0.38	[15]
Fumarate	1.3	[16]
Isocitrate	0.038	[17]
Malate	4	[13]
NAD^+^	2.6	[11]
NADH	0.083	[14]
Oxalacetate *	0.0061	-
Succinate	0.57	[14]
Succinyl-CoA	0.23	[14]
α-ketoglutarate	0.32	[17]

* As reported elsewhere, the actual concentration of this compound in the cell is below the detection limit [11].

**Table 2:** Parameter variation consequences in the different models of Krebs’ cycle. Green indicates that, after perturbation, SS can be obtained. The red indicates that, after perturbation, SS cannot be obtained (no SS).

	V1 × 5	V1/5	V4 × 5	V4/5	V5 × 5	V5/5	V6 × 5	V6/5	V1–V5 × 5	V1–V5/5
Linear-like										
Closed										
Open										

## Data Availability

Raw data that are not already in the Supplementary can be obtained from the corresponding author upon reasonable request.

## Supplementary Materials

The following supporting information can be downloaded at: https://www.mdpi.com/article/10.3390/biomedicines10051199/s1, Equations; Table S1: Reaction fluxes (in RU) and metabolite concentrations (in mM) in different conditions of a “linear” Krebs’s cycle. Table S2. Reaction fluxes (in RU) and metabolite concentrations (in mM) in different conditions of the closed TAC model. Table S3. Reaction fluxes (in RU) and metabolite concentrations (in mM) in different conditions of the open TAC model.
