# Supplementary material for: Robustness of the Krebs Cycle under Physiological Conditions and in Cancer: New Clues for Evaluating Metabolism-Modifying Drug Therapies
Source: Biomedicines. 2022 May 22;10(5):1199. doi: 10.3390/biomedicines10051199 (PMC9138339; doi:10.3390/biomedicines10051199)
Supplement: Supplementary file 1 [file biomedicines-10-01199-s001.zip › biomedicines-1731360-supplementary.pdf]

Supplementary Material

Robustness of the Krebs cycle under physiological conditions and in cancer. New clues for evaluating metabolism-modifying drug therapies

Rafael Franco[1,2,3]* and Joan Serrano-Marín[2]

[1] CiberNed. Network Center for Neurodegenerative diseases. National Spanish Health Institute Carlos III. Madrid. Spain. (rfranco123@gmail.com)
[2] Molecular Neurobiology laboratory. Department of Biochemistry and Molecular Biomedicine. Universitat de Barcelona. Barcelona. Spain.
[3] School of Chemistry. Universitat de Barcelona. Barcelona. Spain.

CONTENTS

- Equations
- Table S1
- Table S2
- Table S3

EQUATIONS

The citrate synthase reaction was settled as Equation 1 (Eq.1):

$$v1 = \frac{\mathrm{Vmax}*[\mathrm{Oxa}]*[\mathrm{ACoA}]}{\mathrm{Km(Oxa)}*\mathrm{Km(ACoA)}+[\mathrm{Oxa}]*\mathrm{Km(ACoA)}+[ACoA]*\mathrm{Km(Oxa)}+[\mathrm{Oxa}]*[\mathrm{ACoA}]} \qquad \text{(Eq. 1)}$$

The Aconitase reactions was settled as:

$$v2 = \frac{\frac{Vf*[\mathrm{Cit}]}{Km(Cit)} - \frac{Vr*[\mathrm{Isocit}]}{Km(Isocit)}}{1+\frac{[\mathrm{Cit}]}{Km(Cit)}+\frac{[\mathrm{Isocit}]}{Km(Isocit)}} \qquad \text{(Eq. 2)}$$

The Isocitrate DH reaction was settled as:

$$v3 = \frac{\mathrm{Vmax}*[\mathrm{Isocit}]*[\mathrm{NAD+}]}{\mathrm{Km(Isocit)}*\mathrm{Km(NAD+)}+[\mathrm{Isocit}]*\mathrm{Km(NAD+)}+[NAD+]*\mathrm{Km(Isocit)}+[\mathrm{Isocit}]*[\mathrm{NAD+}]} \qquad \text{(Eq. 3)}$$

The Alpha-Ketoglutarate DH was settled as:

$$v4 = \frac{\mathrm{Vmax}*[\alpha\mathrm{KG}]}{\mathrm{Km(Isocit)}*\mathrm{Km(NAD+)}+[\mathrm{Isocit}]*\mathrm{Km(NAD+)}+[NAD+]*\mathrm{Km(Isocit)}+[\mathrm{Isocit}]*[\mathrm{NAD+}]} \qquad \text{(Eq. 4)}$$

The Succinyl-CoA synthetase reaction was settled as:

$$v5 = \frac{\frac{Vf*[\mathrm{SCoA}]}{Km(SCoA)} - \frac{Vr*[\mathrm{Suc}]}{Km(Suc)}}{1+\frac{[\mathrm{SCoA}]}{Km(SCoA)}+\frac{[\mathrm{Suc}]}{Km(Suc)}} \qquad \text{(Eq. 5)}$$

The Succinate DH reaction was settled as:

$$v6 = \frac{\frac{Vf*[\mathrm{Suc}]}{Km(Suc)} - \frac{Vr*[\mathrm{Fum}]}{Km(Fum)}}{1+\frac{[\mathrm{Suc}]}{Km(Suc)}+\frac{[\mathrm{Fum}]}{Km(Fum)}*1+\frac{[\mathrm{Fum}]}{Ki(Fum)}} \qquad \text{(Eq. 6)}$$

The Fumarate Hydratase reaction was settled as:

$$v7 = \frac{\frac{Vf*[\mathrm{Fum}]}{Km(Fum)} - \frac{Vr*[\mathrm{Mal}]}{Km(Mal)}}{1+\frac{[\mathrm{Fum}]}{Km(Fum)}+\frac{[\mathrm{Mal}]}{Km(Mal)}} \qquad \text{(Eq. 7)}$$

The Malate DH reaction was settled as:

$$v8 = \frac{Vf*[mal]*[NAD+]-\frac{[Oxa]*[NADH]}{Keq}}{[mal]*[NAD+]+Km(NAD+)*[mal]+[NAD+]*1+\frac{[NADH]}{Ki(NADH)}+\frac{Vf}{Vf*\frac{Keq}{0.046}}*Km(NADH)*[Oxa]*1+\frac{[mal]}{Ki(mal)}+[NADH]*(Km(oxa)+[oxa])} \quad \text{(Eq. 8)}$$

The input reaction was settled as:

$$v9 = \frac{(Vmax*[glut]*[NAD+])}{Km(glut)*Km(NAD+)+[glut]*Km(NAD+)+[NAD+]*Km(glut)+[glut]*[NAD+]} \quad \text{(Eq. 9)}$$

The output reaction was settled as:

$$v10 = \frac{(Vmax*[cit]*[NAD+])}{Km(cit)*Km(NAD+)+[cit]*Km(NAD+)+[NAD+]*Km(cit)+[cit]*[NAD+]} \quad \text{(Eq. 10)}$$

Table S1. Reaction fluxes (in RU) and metabolite concentrations (in mM) in different conditions of a “linear” Krebs’s cycle. The fluxes of each enzyme are indicated for the different conditions. In the first column is indicated the Basal (intial conditions), later the following abbreviations have been used. V1: citrate synthase (CS); V2; Aconitase (Aco); V3 isocitrate dehydrogenase (IDH); V4: α-ketoglutarate dehydrogenase (αKDH); V5: succinyl-CoA synthetase (SuS);V6: succinate dehydrogenase; (SDH); V7: fumarase (FH); V8: malate dehydrogenase (MDH): Intermediates: cit: citrate; akg: α-ketoglutarate; suc: succinate.

Fluxes through every step																					
	Enzyme activity variation												Concentration variation								
	**Control**	V1 x5	V1 /5	V4 x5	V4 /5 (a)	V5 x5	V5 /5 (a)	V6 x5	V6 /5	(V1,5) x5	(V1,5) /5 (a)	(V4,6) x5 (a)	cit x2	cit /2	akg x2	akg /2	akg x100	akg /100	suc x100	suc /100	(akg, cit) x2	(akg, cit) /2 (a)
CS	100.00	500.00	20.00	100.00	100.00	100.00	100.00	100.00	100.00	500.00	20.00	100.00	100.00	100.00	100.00	100.00	100.00	100.00	100.00	100.00	100.00	100.00
Aco	100.00	100.00	100.00	100.00	100.00	100.00	100.00	100.00	100.00	100.00	100.00	100.00	131.85	70.70	100.00	100.00	100.00	100.00	100.00	100.00	131.85	70.70
IDH	100.00	100.00	100.00	100.00	100.00	100.00	100.00	100.00	100.00	100.00	100.00	100.00	131.85	70.70	100.00	100.00	100.00	100.00	100.00	100.00	131.85	70.70
αKDH	100.00	100.00	100.00	100.00	32.50	100.00	100.00	100.00	100.00	100.00	100.00	100.00	131.85	70.70	100.00	100.00	100.00	100.00	100.00	100.00	131.85	70.70
SuS	100.00	100.00	100.00	100.00	32.50	100.00	37.82	100.00	100.00	100.00	37.82	100.00	123.80	70.70	100.00	100.00	100.00	100.00	100.00	100.00	123.80	70.70
SDH	100.00	100.00	100.00	100.00	32.50	100.00	37.82	100.00	100.00	100.00	37.82	100.00	102.80	70.70	100.00	100.00	100.00	100.00	100.00	100.00	102.80	70.70
FH	100.00	100.00	100.00	100.00	32.50	100.00	37.82	100.00	100.00	100.00	37.82	100.00	102.80	70.70	100.00	100.00	100.00	100.00	100.00	100.00	102.80	70.70
MDH	100.00	100.00	100.00	100.00	32.50	100.00	37.82	100.00	100.00	100.00	37.82	100.00	102.80	70.70	100.00	100.00	100.00	100.00	100.00	100.00	102.80	70.70

Intermediate concentrations																					
	Enzyme activity variation												Concentration variation								
	**Control**	V1 x5	V1 /5	V4 x5	V4 /5 (a)	V5 x5	V5 /5 (a)	V6 x5	V6 /5	(V1,5) x5	(V1,5) /5 (a)	(V4,6) x5	cit x2 (a)	cit /2	akg x2	akg /2	akg x100	akg /100	Suc x100	Suc /100	(akg, cit) x2 (a)	(akg, cit) /2 (a)
[icit]	0.04	0.04	0.04	0.04	0.04	0.04	0.04	0.04	0.04	0.04	0.04	0.04	0.05	0.03	0.04	0.04	0.04	0.04	0.04	0.04	0.05	0.03
[akg]	0.32	0.32	0.32	0.03	67501.40	0.32	0.32	0.32	0.32	0.32	0.32	0.03	0.86	0.15	0.32	0.32	0.32	0.32	0.32	0.32	0.86	0.15
[sca]	0.23	0.23	0.23	0.23	0.02	0.03	62178.2	0.13	1.02	0.03	62178.20	0.13	8047.89	0.05	0.23	0.23	0.23	0.23	0.23	0.23	8047.97	0.05
[suc]	0.57	0.57	0.57	0.57	0.001	0.57	0	0.15	3.96	0.57	0.001	0.15	20986.80	0.004	0.57	0.57	0.57	0.57	0.57	0.57	20987.00	0.004
[fum]	1.30	1.30	1.30	1.30	0.01	1.3	0.01	1.3	1.3	1.30	0.01	1.30	16.53	0.04	1.30	1.30	1.30	1.30	1.30	1.30	16.53	0.04
[mal]	4.00	4.00	4.00	4.00	0.09	4	0.11	4	4	4.00	0.11	4.00	9.36	0.41	4.00	4.00	4.00	4.00	4.00	4.00	9.36	0.41
[oxa]	0.01	0.01	0.01	0.01	0.01	0.01	0.01	0.01	0.01	0.01	0.01	0.01	0.01	0.01	0.01	0.01	0.01	0.01	0.01	0.01	0.01	0.01

(a) no SS is reached in these conditions

Table S2. Reaction fluxes (in RU) and metabolite concentrations (in mM) in different conditions of the closed TAC model. The fluxes of each enzyme are indicated for the different conditions. In the first column is indicated the Basal (initial conditions), later the following abbreviations have been used. V1: citrate synthase; V4: α-ketoglutarate dehydrogenase; V5: succinyl-CoA synthetase ;V6: succinate dehydrogenase; cit: citrate; αkg: α-Ketoglutarate; suc: succinate.

Fluxes through every step																					
	Enzyme activity variation												Concentration variation								
	**Control**	V1 x5	V1 /5	V4 x5	V4 /5	V5 x5	V5 /5	V6 x5	V6 /5	(V1,5) x5	(V1,5) /5	(V4,6) x5	cit x2	cit /2	akg x2	akg /2	akg x100	akg /100	Suc x100	Suc /100	(akg, cit) x2	(akg, cit) /2
CS	100.00	101.60	29.70	100.26	31.50	100.19	37.3	100.63	98.11	101.71	29.61	100.90	100.33	99.80	100.28	99.84	102.64	99.66	102.77	99.34	100.55	99.62
Aco	100.00	101.60	29.70	100.26	31.50	100.19	37.3	100.63	98.11	101.71	29.61	100.90	100.33	99.80	100.28	99.84	102.64	99.66	102.77	99.34	100.55	99.62
IDH	100.00	101.60	29.70	100.26	31.50	100.19	37.3	100.63	98.11	101.71	29.61	100.90	100.33	99.80	100.28	99.84	102.64	99.66	102.77	99.34	100.55	99.62
αKDH	100.00	101.60	29.70	100.26	31.50	100.19	37.3	100.63	98.11	101.71	29.61	100.90	100.33	99.80	100.28	99.84	102.64	99.66	102.77	99.34	100.55	99.62
SuS	100.00	101.60	29.70	100.26	31.50	100.19	37.3	100.63	98.11	101.71	29.61	100.90	100.33	99.80	100.28	99.84	102.64	99.66	102.77	99.34	100.55	99.62
SDH	100.00	101.60	29.70	100.26	31.50	100.19	37.3	100.63	98.11	101.71	29.61	100.90	100.33	99.80	100.28	99.84	102.64	99.66	102.77	99.34	100.55	99.62
FH	100.00	101.60	29.70	100.26	31.50	100.19	37.3	100.63	98.11	101.71	29.61	100.90	100.33	99.80	100.28	99.84	102.64	99.66	102.77	99.34	100.55	99.62
MDH	100.00	101.60	29.70	100.26	31.50	100.19	37.3	100.63	98.11	101.71	29.61	100.90	100.33	99.80	100.28	99.84	102.64	99.66	102.77	99.34	100.55	99.62

Intermediate concentrations																					
	Enzyme activity variation												Concentration variation								
	**Control**	V1 x5	V1 /5	V4 x5	V4 /5	V5 x5	V5 /5	V6 x5	V6 /5	(V1,5) x5	(V1,5) /5	(V4,6) x5	cit x2	cit /2	akg x2	akg /2	akg x100	akg /100	Suc x100	Suc /100	(akg, cit) x2	(akg, cit) /2
[cit]	0.38	0.39	0.05	0.38	0.05	0.38	0.07	0.39	0.36	0.39	0.05	0.39	0.38	0.38	0.38	0.38	0.40	0.38	0.40	0.37	0.38	0.38
[icit]	0.04	0.04	0.01	0.04	0.01	0.04	0.01	0.04	0.04	0.04	0.01	0.04	0.04	0.04	0.04	0.04	0.04	0.04	0.04	0.04	0.04	0.04
[αkg]	0.32	0.33	0.04	0.03	6.29	0.32	0.06	0.33	0.3	0.33	0.04	0.03	0.32	0.32	0.32	0.32	0.34	0.32	0.34	0.31	0.32	0.32
[sca]	0.23	0.39	0.02	0.26	0.02	0.04	6.11	0.15	0.37	0.07	0.31	0.16	0.26	0.21	0.26	0.22	5.32	0.20	9.90	0.19	0.30	0.20
[suc]	0.57	1.20	0.002	0.68	0.001	0.65	0.001	0.22	1.23	1.38	0.002	0.26	0.71	0.51	0.69	0.52	21.16	0.46	39.61	0.39	0.83	0.45
[fum]	1.30	1.91	0.03	1.42	0.01	1.39	0.01	1.64	0.78	2.04	0.03	1.84	1.46	1.22	1.43	1.23	6.87	1.17	8.57	1.06	1.59	1.15
[mal]	4.00	2.58	6.64	4.04	0.46	4.03	0.58	4.09	3.76	2.58	6.35	4.12	4.04	3.97	4.04	3.98	4.38	3.95	4.40	3.91	4.07	3.95
[oxa]	0.01	0.001	0.05	0.01	0.001	0.01	0.001	0.01	0.01	0.001	0.05	0.01	0.01	0.01	0.01	0.01	0.01	0.01	0.01	0.01	0.01	0.01

Table S3. Reaction fluxes (in RU) and metabolite concentrations (in mM) in different conditions of the open Krebs' cycle model. The fluxes of each enzyme are indicated for the different conditions. In the first column is indicated the Basal (initial conditions), later the following abbreviations have been used. V1: citrate synthase; V4: α-ketoglutarate dehydrogenase; V5: succinyl-CoA synthetase ;V6: succinate dehydrogenase; cit: citrate; αkg: α-ketoglutarate; suc: succinate.

Fluxes through every step																						
	Enzyme activity variation												Concentration variation									
	**Control**	V1 x5	V1 /5 (a)	V4 x5	V4 /5 (a)	V5 x5	V5 /5 (a)	V6 x5	V6 /5	(V1,5) x5	(V1,5) /5 (a)	(V4,6) x5	cit x2	cit /2	akg x2	akg /2	akg x100	akg /100	Suc x100	Suc /100	(akg, cit) x2	(akg, cit) /2
CS	100.00	100.00	31.79	100.00	32.20	100.00	37.82	100.00	100.00	100.00	31.79	100.00	100.00	100.00	100.00	100.00	100.00	100.00	100.00	100.00	100.00	100.00
Aco	80.00	80.00	27.86	80.00	28.20	80.00	32.88	80.00	80.00	80.00	27.85	80.00	80.00	80.00	80.00	80.00	80.00	80.00	80.00	80.00	80.00	80.00
IDH	80.00	80.00	27.86	80.00	28.20	80.00	32.88	80.00	80.00	80.00	27.85	80.00	80.00	80.00	80.00	80.00	80.00	80.00	80.00	80.00	80.00	80.00
αKDH	100.00	100.00	47.86	100.00	32.20	100.00	52.88	100.00	100.00	100.00	47.85	100.00	100.00	100.00	100.00	100.00	100.00	100.00	100.00	100.00	100.00	100.00
SuS	100.00	100.00	46.66	100.00	32.20	100.00	37.82	100.00	100.00	100.00	37.68	100.00	100.00	100.00	100.00	100.00	100.00	100.00	100.00	100.00	100.00	100.00
SDH	100.00	100.00	31.79	100.00	32.20	100.00	37.82	100.00	100.00	100.00	37.12	100.00	100.00	100.00	100.00	100.00	100.00	100.00	100.00	100.00	100.00	100.00
FH	100.00	100.00	31.79	100.00	32.20	100.00	37.82	100.00	100.00	100.00	37.09	100.00	100.00	100.00	100.00	100.00	100.00	100.00	100.00	100.00	100.00	100.00
MDH	100.00	100.00	31.79	100.00	32.20	100.00	37.82	100.00	100.00	100.00	31.79	100.00	100.00	100.00	100.00	100.00	100.00	100.00	100.00	100.00	100.00	100.00
Output	20.00	20.00	3.93	20.00	4.00	20.00	4.94	20.00	20.00	20.00	3.93	20.00	20.00	20.00	20.00	20.00	20.00	20.00	20.00	20.00	20.00	20.00
Input	20.00	20.00	20.00	20.00	20.00	20.00	20.00	20.00	20.00	20.00	20.00	20.00	20.00	20.00	20.00	20.00	20.00	20.00	20.00	20.00	20.00	20.00

Intermediate concentrations																						
	Enzyme activity variation												Concentration variation									
	**Control**	V1 x5	V1 /5 (a)	V4 x5	V4 /5 (a)	V5 x5	V5 /5 (a)	V6 x5	V6 /5	(V1,5) x5	(V1,5) /5 (a)	(V4,6) x5	cit x2	cit /2	akg x2	akg /2	akg x100	akg /100	Suc x100	Suc /100	(akg, cit) x2	(akg, cit) /2
[cit]	0.38	0.38	0.06	0.38	0.06	0.38	0.08	0.38	0.38	0.38	0.06	0.38	0.38	0.38	0.38	0.38	0.38	0.38	0.38	0.38	0.38	0.38
[icit]	0.04	0.04	0.01	0.04	0.01	0.04	0.02	0.04	0.04	0.04	0.01	0.04	0.04	0.04	0.04	0.04	0.04	0.04	0.04	0.04	0.04	0.04
[αkg]	0.32	0.32	0.08	0.03	16005.70	0.32	0.10	0.32	0.32	0.32	0.08	0.03	0.32	0.32	0.32	0.32	0.32	0.32	0.32	0.32	0.32	0.32
[sca]	0.23	0.17	11009.80	0.23	0.02	0.03	15067.30	0.13	1.02	0.02	10077.50	0.13	0.23	0.23	0.23	0.23	0.23	0.23	0.23	0.23	0.23	0.23
[suc]	0.57	0.32	136296.00	0.57	0.001	0.57	0.001	0.15	3.96	0.32	191.45	0.15	0.57	0.57	0.57	0.57	0.57	0.57	0.57	0.57	0.57	0.57
[fum]	1.30	0.95	53.63	1.30	0.01	1.30	0.01	1.30	1.30	0.95	28.28	1.30	1.30	1.30	1.30	1.30	1.30	1.30	1.30	1.30	1.30	1.30
[mal]	4.00	2.47	13308.90	4.00	0.47	4.00	0.59	4.00	4.00	2.47	5771.47	4.00	4.00	4.00	4.00	4.00	4.00	4.00	4.00	4.00	4.00	4.00
[oxa]	0.01	0.001	7.85	0.01	0.001	0.01	0.001	0.01	0.01	0.001	7.15	0.01	0.01	0.01	0.01	0.01	0.01	0.01	0.01	0.01	0.01	0.01

(a) no SS is reached in these condition
